# Sauna dehydration as a new physiological challenge model for intestinal barrier function

**DOI:** 10.1038/s41598-021-94814-0

**Published:** 2021-07-30

**Authors:** Maria Fernanda Roca Rubio, Ulrika Eriksson, Robert J. Brummer, Julia König

**Affiliations:** 1 Nutrition-Gut-Brain Interactions Research Centre, Faculty of Medicine and Health, School of Medical Sciences, Örebro University, 701 82 Örebro, Sweden; 2 Man-Technology-Environment (MTM) Research Centre, School of Science and Technology, Örebro University, 701 82 Örebro, Sweden

**Keywords:** Physiology, Medical research

## Abstract

The intestinal barrier plays a crucial role in maintaining gut health, and an increased permeability has been linked to several intestinal and extra-intestinal disorders. There is an increasing demand for interventions aimed at strengthening this barrier and for in vivo challenge models to assess their efficiency. This study investigated the effect of sauna-induced dehydration on intestinal barrier function (clinicaltrials.gov: NCT03620825). Twenty healthy subjects underwent three conditions in random order: (1) Sauna dehydration (loss of 3% body weight), (2) non-steroidal anti-inflammatory drug (NSAID) intake, (3) negative control. Intestinal permeability was assessed by a multi-sugar urinary recovery test, while intestinal damage, bacterial translocation and cytokines were assessed by plasma markers. The sauna dehydration protocol resulted in an increase in gastroduodenal and small intestinal permeability. Presumably, this increase occurred without substantial damage to the enterocytes as plasma intestinal fatty acid-binding protein (I-FABP) and liver fatty acid-binding protein (L-FABP) were not affected. In addition, we observed significant increases in levels of lipopolysaccharide-binding protein (LBP), IL-6 and IL-8, while sCD14, IL-10, IFN-ɣ and TNF-α were not affected. These results suggest that sauna dehydration increased intestinal permeability and could be applied as a new physiological in vivo challenge model for intestinal barrier function.

## Introduction

The intestinal barrier is a physical and functional barrier that prevents translocation of harmful substances while allowing a peaceful coexistence with intestinal symbionts^1^. Dysfunction of the gut barrier can lead to increased intestinal permeability. Several gastrointestinal and extraintestinal disorders have been linked to an increased intestinal permeability, such as inflammatory bowel diseases (IBD), irritable bowel syndrome (IBS), coeliac disease, food allergies, and metabolic syndrome, amongst others^1^. A functional intestinal barrier is a pivotal component in maintaining optimal gut health and plays an important role in the gut-brain axis. Hence, several nutritional interventions in general and the use of pre- and probiotics specifically are intended to strengthen the gut barrier function. In order to assess the efficiency of such interventions, the barrier function has to be challenged and so far, generally applicable and valid in vivo challenge models of intestinal barrier function are scarce.

Strenuous exercise is a well-established model to increase intestinal permeability^2–5^. However, exercise studies only include well-trained athletes as the intensity needed to induce increased permeability is rather high (e.g., 60 min running at 80% of V0_2_ max) and is difficult to achieve for untrained individuals^3,6^. Moreover, it is known that untrained and trained individuals differ when it comes to body composition, functional capacity^7^ and thermotolerance^8^. Hence, the results observed in trained subjects cannot necessarily be extrapolated to the rest of the population.

Previous studies have shown that exercise-induced intestinal permeability is enhanced by both dehydration^4,9,10^ and increased body temperature^11–13^. Therefore, the combination of these two factors might be sufficient to induce intestinal permeability and consequently serve as a potential new model to challenge the intestinal barrier function even in untrained individuals. It was therefore the aim of this study to explore the effect of heat-induced dehydration at rest on small and large intestinal barrier function. For this purpose, we applied a sauna dehydration protocol. Sauna bathing is well tolerated and generally safe for healthy individuals as well as for most patients with stable coronary heart disease^14–16^.

Intestinal permeability was assessed by a multi-sugar urinary recovery test of orally administered water-soluble, non-metabolizable sugars that differ in size. This is a sensitive, non-invasive method capable to detect small changes in small and large intestinal permeability. In this test, the larger sugar molecules, such as lactulose and sucralose can only cross the intestinal barrier by paracellular passage and are not taken up actively^17^. The smaller molecules, such as rhamnose and erythritol, cross the epithelial barrier transcellularly and act as a control for gastric emptying, dilution, transit time, and epithelial absorptive area, as well as systemic distribution and renal function. The urinary recovery ratio is then used as a standardized assessment of the intestinal permeability of the intestinal segment where the permeability probes are absorbed^1,18–20^. The urinary sucrose recovery in fraction 1 (0–5 h) was used as an indicator of gastroduodenal permeability, the lactulose/rhamnose (L/R) ratio in fraction 1 (0–5 h) was used as an indicator of small intestinal permeability, and the sucralose/erythritol (S/E) ratio in fraction 2 (5–24 h) was used as an indicator of colonic permeability^21^*.* In addition, we assessed intestinal and liver fatty acid-binding proteins (I-FABP and L-FABP) in plasma as markers of intestinal damage^1,2,22–27^. These proteins are particularly expressed in cells present on the tips of the villi and released into the blood stream upon enterocyte damage^1,2,22–27^. Plasma concentrations of lipopolysaccharide-binding protein (LBP) and soluble CD14 (sCD14) were assessed as a marker of translocation of bacterial products into the blood stream^28,29^. Immune response elicited from the sauna dehydration was assessed by the concentration of plasma cytokines IL-6, IL-8, IL-10, IFN-ɣ, TNF-α.

To account for individual variations of these markers and to improve statistical power, we applied a crossover design where all subjects participated in three visits in random order: negative control, sauna dehydration, and positive control (intake of indomethacin). Indomethacin, a nonsteroidal anti-inflammatory drug (NSAID), has repeatedly shown to increase intestinal permeability assessed by the multi-sugar test in healthy subjects^17,30–32^ and allowed for comparison of the results from the sauna visit to an established inducer of intestinal permeability.

The results of this study suggested that the combination of dehydration and heat successfully increased intestinal permeability. Sauna dehydration could therefore be considered as a new and widely applicable physiological challenge model for intestinal barrier function.

## Results

### Subject characteristics

The study was performed at Örebro University in Örebro, Sweden, from March 2018 to June 2018. Twenty healthy subjects (10 females and 10 males; mean age 26.7 ± 5.1 years) were included in this study. Baseline characteristics of all participants are shown in Table 1. Two participants (one male and one female) only reached a dehydration corresponding to 2% body weight loss during the sauna exposure. As the data of these two participants followed the same pattern as the rest of the study group, their data was included in the analyses.

**Table 1:** Baseline characteristics of study participants.

Characteristics	
Female/Male	10/10
Age (years, mean ± SD)	26.7 ± 5.1
Body mass index (kg/m^2^, mean ± SD)	23.1 ± 3.0
Total time* until loss of 1.5% body weight (h: min, mean ± SD)	1:48 ± 0:23
Total time* until loss of 3% body weight (h: min, mean ± SD)	3:29 ± 0:37

* Total time refers to the total time needed to reach the respective dehydration, including both sauna and cooling down periods.

### Tympanic temperature during sauna exposure

Tympanic temperature was measured as an indicator of core body temperature^33^ during the sauna visit and registered at three different time points; after 15 min in the sauna, when reaching 1.5% dehydration and at 3% dehydration. Friedman’s test showed no significant difference among the chosen time points (*P* = 0.291, n = 13). Median tympanic temperature after 15 min in the sauna was 38.0 °C (IQR 36.2–38.9 °C), 38.8 °C (38.0–39.7 °C) at 1.5% dehydration and 38.6 °C (37.4–39.1 °C) at 3% dehydration, data not shown. Due to technical difficulties, data of seven participants are missing from all these analyses*.*

### Effect of sauna dehydration on gastroduodenal permeability

**Figure 1:**
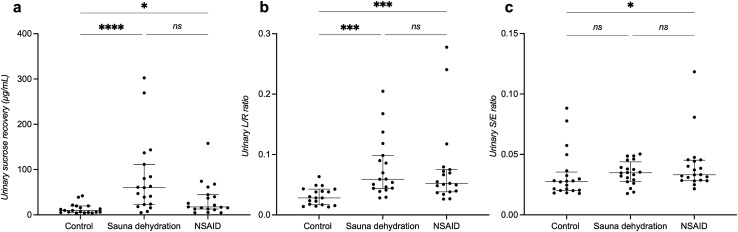
Intestinal permeability at the different test conditions. (**a**) Gastroduodenal permeability measured by urinary sucrose recovery (0-5 h). (**b**) Small intestinal permeability measured by lactulose/rhamnose (L/R) excretion ratio (0-5 h). (**c**) Colonic permeability measured by urinary sucralose/erythritol (S/E) excretion ratio (5-24 h). The horizontal line marks the median, the vertical line spans through the interquartile range (IQR). The dots represent the individuals.*P < 0.05. **P < 0.01. ***P < 0.001. ****P < 0.0001. ns, non-significant. NSAID, nonsteroidal anti-inflammatory drug.

Gastroduodenal permeability was measured by urinary sucrose recovery (0–5 h). Friedman’s test showed a significant difference among the test conditions (*P* < 0.001, n = 19). Gastroduodenal permeability was significantly increased after the sauna dehydration (median of 54.0 µg/ml, interquartile range [IQR]: 23.1–104.6 µg/ml compared to the control condition (10.0 µg/ml, 5.5–20 µg/ml, *P* < 0.0001, Dunn’s multiple comparison test) (Fig. 1a). A significant increase in gastroduodenal permeability compared to control was also observed after the NSAID intervention (17.5, 12.9–44.6 µg/ml, *P* < 0.05). There was no significant difference in gastroduodenal permeability after the sauna dehydration in comparison to the NSAID intervention (*P* = 0.31). Data of one participant was excluded from all these analyses due to errors in 0–5 h urine sample collection.

### Effect of sauna dehydration on small intestinal permeability

Small intestinal permeability was measured by urinary lactulose/rhamnose (L/R) excretion ratio (0–5 h). Friedman’s test showed a significant difference among the test conditions (*P* < 0.0001, n = 19). Small intestinal permeability was significantly increased after the sauna dehydration (median of 0.059, IQR: 0.044–0.098) compared to the control visit (0.028, 0.017–0.043, *P* < 0.001) at the sauna dehydration (Fig. 1b). A significant increase in small intestinal permeability was also observed after the NSAID intervention compared to the control visit (0.052, 0.038–0.075, *P* < 0.001). There was no significant difference in urinary L/R ratios when comparing sauna dehydration to NSAID intervention (*P* > 0.99). Data of one participant was excluded from all these analyses due to errors in 0–5 h urine sample collection.

### Effect of sauna dehydration on colonic permeability

Colonic permeability was measured as the urinary sucralose/erythritol (S/E) excretion ratio (5–24 h). Friedman’s test showed a significant difference in colonic permeability among the test conditions (*P* < 0.05). Sauna dehydration did not result in a significant increase in colonic permeability (Fig. 1c; control visit: median of 0.027, IQR: 0.020–0.035; sauna dehydration: 0.035, IQR: 0.028–0.044; *P* = 0.17). There was a significant increase in urinary S/E ratio after the NSAID intervention (0.033, 0.028–0.045) compared to the control visit (*P* < 0.05). There was no significant difference in urinary S/E ratios when comparing sauna dehydration to NSAID intervention (*P* > 0.99).

### Effect of sauna dehydration on markers of intestinal damage

#### Intestinal fatty acid-binding protein (I-FABP)

**Figure 2:**
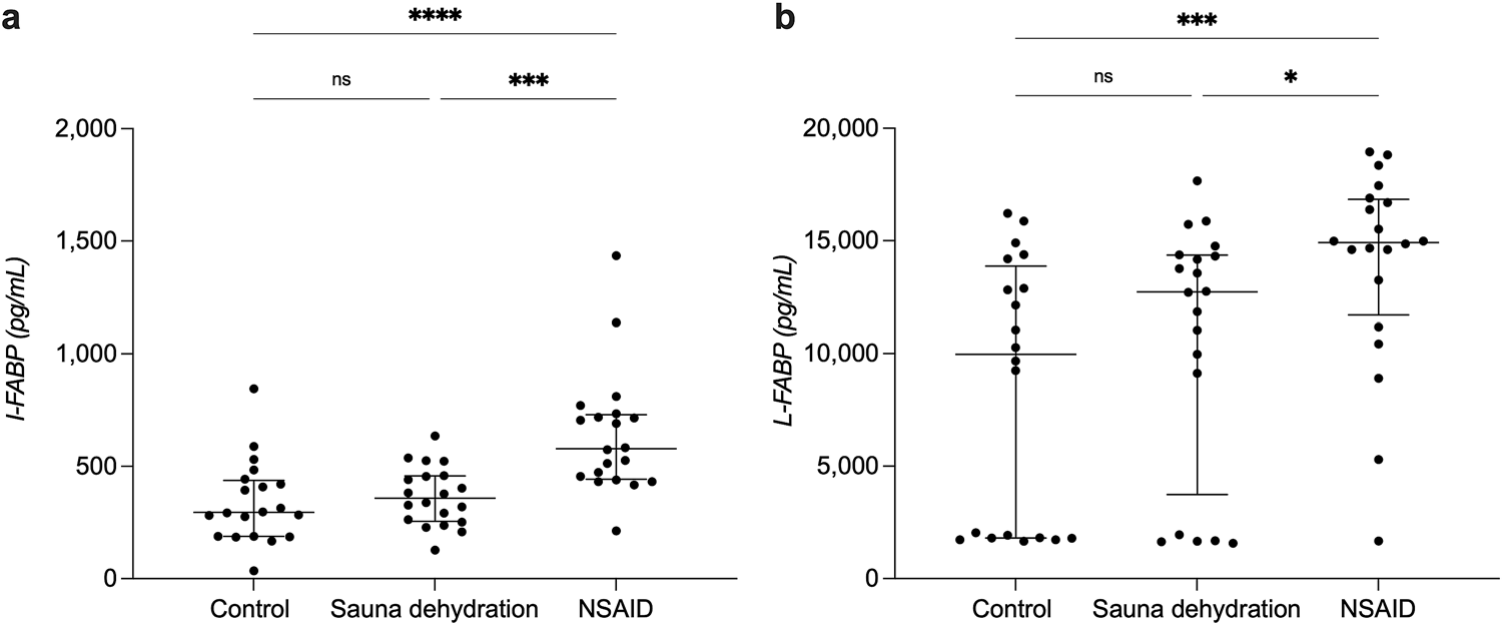
Plasma fatty acid-binding proteins concentrations at the different test conditions. (**a**) Plasma concentrations of intestinal fatty acid-binding protein (I-FABP). (**b**) Plasma concentrations of liver fatty acid-binding protein (L-FABP). The horizontal line marks the median, the vertical line spans through the interquartile range (IQR). The dots represent the individuals. *P < 0.05. ****P < 0.0001. ns, non-significant. NSAID, nonsteroidal anti-inflammatory drug.

Friedman’s test showed a significant difference in I-FABP concentrations among the test conditions (*P* < 0.0001). The NSAID intervention resulted in a significant increase in I-FABP concentrations (median of 578 pg/ml, IQR: 443–730 pg/ml) in comparison to both control condition (295 pg/ml, 188–437 pg/ml, *P* < 0.0001) as well as sauna condition (358 pg/ml, 255–458 pg/ml, *P* < 0.001). There was no significant difference in plasma I-FABP concentrations after sauna dehydration compared to the control condition (*P* > 0.99) (Fig. 2a).

#### Liver fatty acid-binding protein (L-FABP)

Friedman’s test showed a significant difference in L-FABP concentrations among the test conditions (*P* = 0.001). The NSAID intervention resulted in a significant increase in L-FABP concentrations (median of 14,922 pg/ml, IQR: 11,701–16,843 pg/ml) in comparison to both control condition (9,977 pg/ml, 1,805–13,869 pg/ml, *P* < 0.001) and sauna dehydration condition (12,729 pg/ml, 3,742–14,360 pg/ml, *P* < 0.05). There was no significant difference in plasma L-FABP concentrations after sauna dehydration compared to the control condition (*P* = 0.80) (Fig. 2b).

### Effect of sauna dehydration on markers of bacterial translocation

#### Lipopolysaccharide-binding protein (LBP)

**Figure 3:**
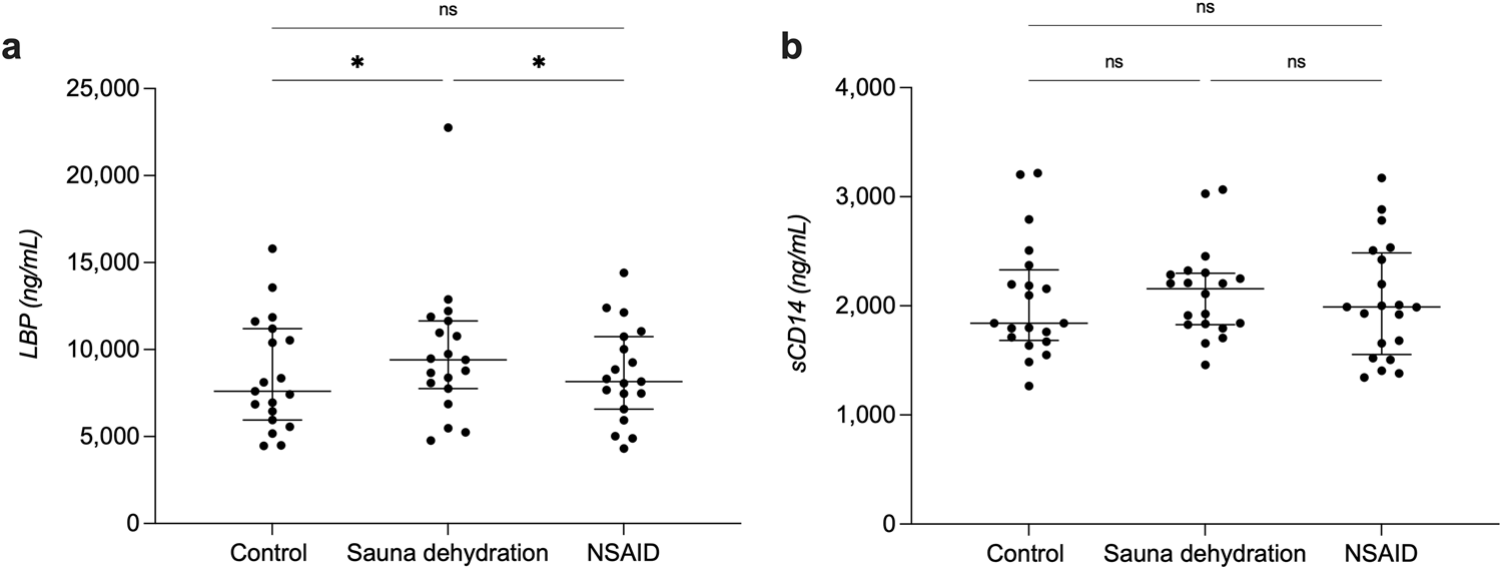
Lipopolysaccharide-binding protein and soluble CD14 concentrations at the different test conditions. (**a**) Plasma concentrations of lipopolysaccharide-binding protein (LBP). (**b**) Plasma concentrations of soluble CD14 (sCD14). The horizontal line marks the median, the vertical line spans through the interquartile range (IQR). The dots represent the individuals. * *P* < 0.05. ns, non-significant. NSAID, nonsteroidal anti-inflammatory drug. Data of one participant was excluded from the LBP analysis due to disproportionately high baseline values.

Plasma LBP concentrations were significantly different among the test conditions (*P* < 0.01, Friedman’s test). The sauna dehydration resulted in a significant increase in LBP concentrations from a median of 7,604 ng/ml (IQR 5,947–11,198 ng/ml) at the control condition to a median of 9,407 ng/ml (7,752–11,644 ng/ml, *P* < 0.05) after sauna dehydration (Fig. 3a). The NSAID intervention did not significantly affect LBP concentrations (8,159 ng/ml, 6,576–10,748 ng/ml) in comparison to the control condition (*P* > 0.99). LBP concentrations were significantly increased after the sauna dehydration in comparison to the NSAID intervention (*P* < 0.05). Data of one participant was excluded from the analysis due to disproportionately high baseline values.

#### Soluble CD14 (sCD14)

Plasma sCD14 concentrations were not significantly different among the test conditions (*P* = 0.34, Friedman’s test), with a median of 1,841 ng/ml (IQR 1,682–2,329 ng/ml) at the control condition, 2,157 ng/ml (1,829–2,299 ng/ml) after sauna dehydration and 1,989 ng/ml (1,555–2,486 ng/ml) after the NSAID intervention (Fig. 3b).

### Effect of sauna dehydration on plasma interleukin-6 (IL-6), interleukin-8 (IL-8), interleukin-10 (IL-10), interferon gamma (IFN-ɣ) and tumour necrosis factor alpha (TNF-α)

**Figure 4:**
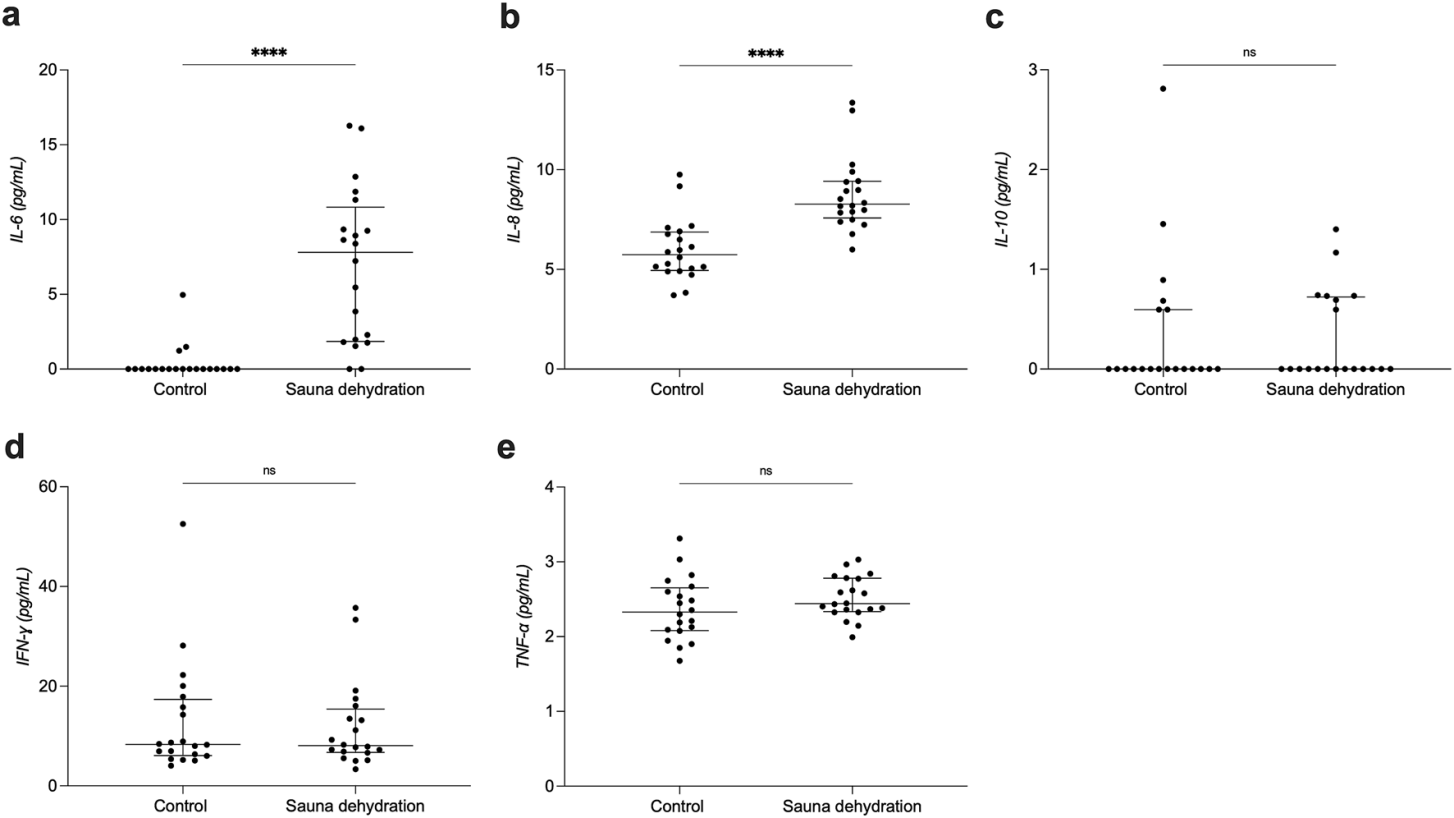
Plasma cytokine concentrations at the control condition and after sauna dehydration. (**a**) IL-6. (**b**) IL-8. (**c**) IL-10. (**d**) IFN-ɣ. (**e**) TNF-α. The horizontal line marks the median, the vertical line spans through the interquartile range (IQR). The dots represent the individuals. ***P < 0.001. ****P < 0.0001. ns, non-significant.

The sauna dehydration resulted in a significant increase in IL-6 concentrations (control visit: median of 0.0 pg/ml, IQR 0.0–0.0 pg/ml; after sauna dehydration: 7.8 pg/ml, 1.9–10.8 pg/ml, *P* < 0.0001, Wilcoxon matched-pairs signed rank test) (Fig. 4a). Also IL-8 concentrations were significantly affected by sauna dehydration (control visit: 5.7, 4.9–6.9 pg/ml; after sauna dehydration: 8.3, 7.6–9.4 pg/ml, *P* < 0.0001) (Fig. 4b). Sauna dehydration did not have an effect on plasma concentrations of IL-10 (control visit: 0.0, 0.0–0.6 pg/ml; after sauna dehydration: 0.0, 0.0–0.7 pg/ml, *P* = 0.1) (Fig. 4c). Also, there was no effect of sauna dehydration on plasma concentrations of IFN-ɣ (control: 8.3; 6.1–17.4; after sauna dehydration: 8.1; 6.7–15.4 pg/ml, *P* = 0.30) (Fig. 4d). Furthermore, there was no effect of sauna dehydration on plasma TNF-α concentrations (control: 2.3; 2.1–2.6 pg/ml; after sauna dehydration: 2.4; 2.3–2.8 pg/ml, *P* = 0.1) (Fig. 4e).

### Differences in urine and plasma markers between female and male participants

**Figure 5:**
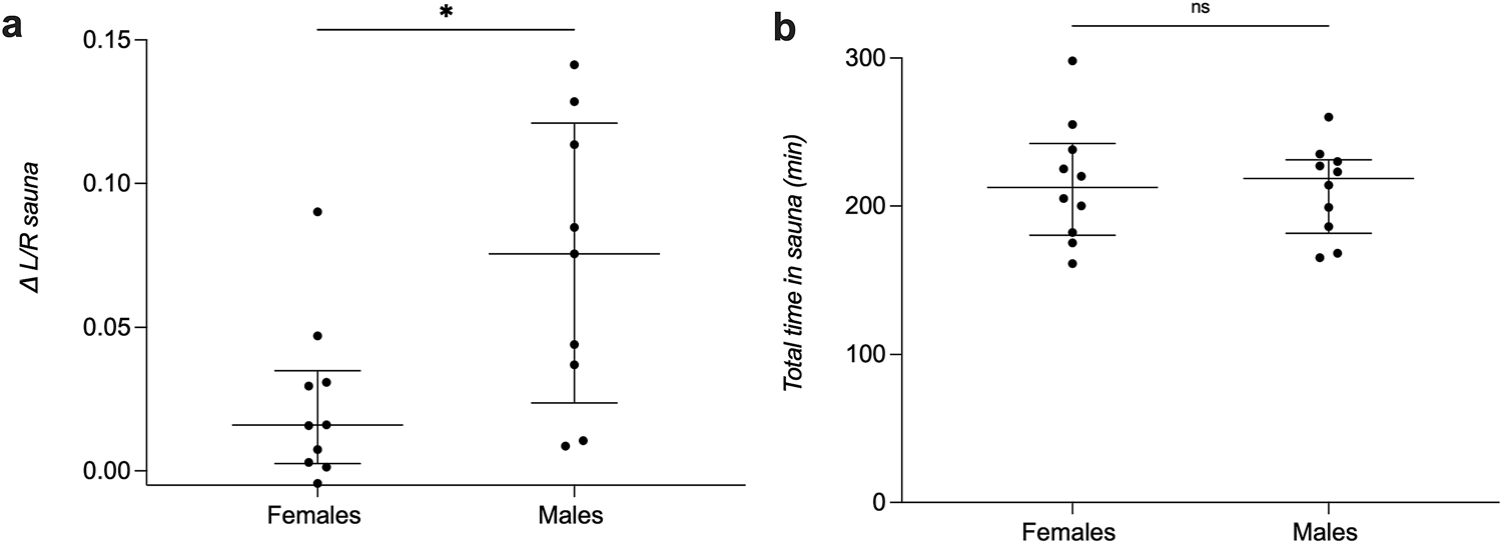
Differences between female and male participants. (**a**) Small intestinal permeability shown as ∆ L/R (L/R sauna–L/R control). (**b**) Total time in sauna until loss of 3% body weight. The horizontal line marks the median, the vertical line spans through the interquartile range (IQR). The dots represent the individuals.*P < 0.05. ns, non-significant.

As the extent of dehydration could be different between females and males due to, for example, body composition, we analysed if there were differences in the effect of the sauna dehydration regarding female and male participants. The increase in small intestinal permeability after sauna compared to the control (∆ L/R [L/R sauna–L/R control]) was significantly higher in males than in females (females: median ∆ L/R of 0.016, IQR 0.002–0.035; males: 0.075, 0.023–0.121, *P* < 0.05, unpaired t test with Welch’s correction, Fig. 5a). No significant differences were found in any of the other markers nor after the intake of the NSAID compared to the control (see Supplementary Fig S1 and Supplementary Fig S2). Also, no significant differences were found in the total time spent in the sauna between female and male participants (females: 213 min, 180–242 min; males: 219; 182–231 min, *P* = 0.75, Fig. 5b) or in their BMI (females: 21.2, 20.2–23.4; males: 24.0, 22.2–26.3, *P* = 0.08).

### Effect of sauna dehydration on salivary cortisol

**Figure 6:**
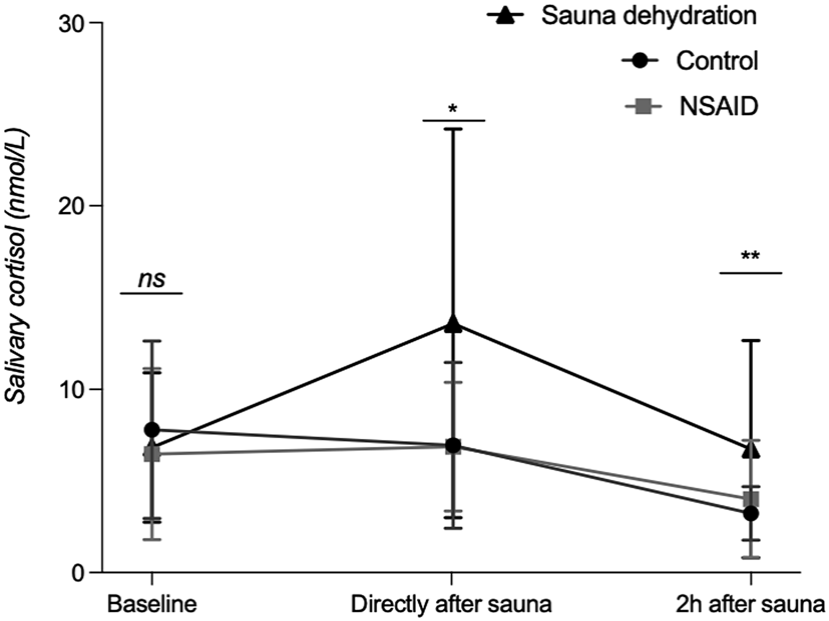
Salivary cortisol concentrations over time during the different test conditions. Saliva samples at the control and NSAID visit were collected at similar times of the day as during the sauna condition in order to consider diurnal changes. Median and IQR are shown. p-values indicate significant differences between the control and sauna dehydration condition. *P < 0.05 (sauna dehydration versus control). **P < 0.01 (sauna dehydration versus control). ns, non-significant. NSAID, nonsteroidal anti-inflammatory drug.

Cortisol responses during the different test conditions are displayed in Fig. 6. The mixed-effects analysis showed a main effect of the test condition (control, sauna dehydration and NSAID) [F (2, 38) = 8.1; *P* < 0.001]. Time of sampling also had a statistically significant effect [F (2, 30) = 8.9; *P* < 0.001]. We also observed a significant condition x time interaction [F (3, 52) = 3.3; *P* < 0.05]. Post-hoc tests (Dunn’s multiple comparisons) showed that there were no significant differences at baseline (control: median of 6.6 nmol/L, interquartile range (IQR) of 4.3–9.9 nmol/L; NSAID: 5.1 nmol/L, 2.8–10.0 nmol/ml; sauna: 7.0 nmol/L, 3.1–9.4 nmol/ml). At 3% dehydration, directly after the sauna protocol, there was a significant increase in cortisol concentrations compared to the control condition (control: 5.7 nmol/L, 3.2–11.0 nmol/L; NSAID: 6.6 nmol/L, 4.1–9.8 nmol/L, *P* = 0.977; sauna: 10.0 nmol/L, 6.3–20.0 nmol/L, adjusted p-value < 0.05). Cortisol concentrations remained significantly increased two hours after the end of the sauna dehydration protocol compared to the control condition (control: 2.9 nmol/L, 2.2–4.2 nmol/L; NSAID: 3.3 nmol/L, 2.5–4.0 nmol/L, *P* = 0.515; sauna: 3.9 nmol/L, 3.2–9.6 nmol/mL, *P* < 0.01).

### Correlations between cortisol levels and increased small intestinal permeability after the sauna condition

As cortisol levels were significantly increased after the sauna dehydration and because stress is known to increase small intestinal permeability, we assessed if cortisol levels and L/R ratios correlated. There was no significant correlation between psychological stress, measured as ∆∆ salivary cortisol [(cortisol at sauna visit, 1 min after—cortisol at sauna visit, 2 h before)—(cortisol at control visit, 1 min after—cortisol at control visit, 2 h before)] and small intestinal permeability, measured as ∆ L/R ratio (L/R ratio at sauna visit-L/R ratio at control visit) (correlation coefficient r = 0.074, *P* = 0.758, data not shown).

### Correlations between body temperature and markers associated with intestinal permeability and cytokines after the sauna condition

As heat is known to increase small intestinal permeability during exercise and can result in increased plasma cytokine levels, we performed correlation analyses to investigate if temperature correlated with markers associated with intestinal permeability (urinary sucrose, L/R, S/E, I-FABP, L-FABP, LBP, sCD14) or plasma cytokines (IL-6, IL-8, IL-10, IFN-ɣ, TNF-α). No correlation was found to be significant after Bonferroni correction for multiple comparison (Supplementary Table S1).

### Correlations between cytokines and markers associated with intestinal permeability after the sauna condition

We performed correlation analyses in order to assess if the increase in some of the plasma cytokines correlated with the L/R ratio after the sauna condition. No correlation was found to be significant after Bonferroni correction for multiple comparison (Supplementary Table S2). In addition, none of the plasma cytokines correlated significantly with any of the plasma markers associated with intestinal permeability (I-FABP, L-FABP, LBP, sCD14) after the sauna condition (Supplementary Table S3).

### Correlations between plasma markers associated with intestinal permeability and small bowel permeability

We performed correlation analyses to investigate if any of the plasma markers correlated with the L/R ratios (i.e., small bowel permeability) and could potentially serve as alternative markers of small intestinal barrier function. However, L/R ratio did not significantly correlate with any of the analysed markers, neither after the sauna dehydration nor after the NSAID intervention (Supplementary Table S4 and S5).

## Discussion

In the current study, we showed that the combination of heat and dehydration at rest as part of a sauna protocol resulted in an increased small intestinal permeability, assessed by the L/R ratio, in healthy subjects. Presumably, this increase occurred without substantial damage to the enterocytes as plasma intestinal fatty acid-binding protein (I-FABP) and liver fatty acid-binding protein (L-FABP) were not affected. We also observed a significant increase in gastroduodenal permeability as well as in levels of lipopolysaccharide-binding protein (LBP), IL-6 and IL-8, while sCD14, IL-10, IFN-ɣ and TNF-α were not affected.

There are different possibilities to increase intestinal permeability in a research setting. In our study, we used a non-steroidal anti-inflammatory drug (NSAID) treatment as a positive control, and indeed, intake of indomethacin increased small intestinal permeability assessed by the multi-sugar test as previously shown^17,30–32^. Apart from NSAIDs, also psychological stress^30,34^ and strenuous endurance exercise^5,6,35,36^ have shown to affect barrier function. The induction of psychological stress in a research setting can be challenging and difficult to standardize, while exercise-induced intestinal permeability can usually only be achieved by well-trained athletes. To the best of our knowledge, our study is the first to show that heat-induced dehydration at rest is sufficient to increase small intestinal permeability.

In our study intestinal permeability was assessed using a non-invasive multi-sugar urinary recovery test. This test allows to detect small changes in small and large intestinal permeability and consists of measuring the urinary recovery of orally administered non-metabolizable sugars^17,37^. Even though the participants were at rest, the combined effect of heat and dehydration produced an increase in small intestinal permeability similar to the one observed during strenuous exercise^5^. Sauna dehydration also increased gastroduodenal permeability, but did not affect large intestinal permeability.

Markers commonly used to assess intestinal damage are plasma I-FABP and L-FABP^1,2,22–27^. It has been previously reported that NSAID intake induces enterocyte damage^17,38–40^, which we also observed in our study. Strenuous endurance exercise studies have consistently reported an increase in small intestinal permeability accompanied by an increase in concentrations of plasma I-FABP, suggesting that exercise-induced intestinal permeability is associated with enterocyte damage^2,5^. A recent study explored the effect of warm (30 °C) and temperate (22 °C) ambient conditions during endurance exercise. Although small intestinal permeability assessed by L/R ratio remained similar among warm and temperate ambient conditions, plasma concentrations of I-FABP were higher in the warm compared to the temperate condition, suggesting that intestinal damage during endurance exercise is enhanced by heat stress^41^. Sheahen et al. studied the effect of passive heat (45 min at 30 °C) on intestinal integrity using I-FABP as a surrogate marker for enterocyte damage. The authors did not find an increase of I-FABP after euhydrated passive heat exposure^13^. As we also did not see an increase in I-FABP or L-FABP, this might suggest that only heat exposure and dehydration, without additional factors such as strenuous exercise or drug intake, do not result in enterocyte damage. In addition, in our study I-FABP and L-FABP showed no correlations with intestinal permeability assessed by the multi-sugar test. These findings highlight the importance of differentiating between enterocyte damage (which I-FABP and L-FABP are a marker of) with associated permeability alteration, and an increase in intestinal permeability per se, which can also be caused by injuring other structures of the intestinal barrier^42^. Hence, even though I-FABP and L-FABP seem to be suitable markers of enterocyte damage, they are not necessarily adequate surrogate markers for intestinal permeability assessed by in vivo tests such as the multi-sugar test.

A result of increased intestinal permeability can be the translocation of bacterial products (such as endotoxins) into the blood stream. Lipopolysaccharides (LPS) are the major cell wall component of gram-negative bacteria. In a healthy intestinal barrier, LPS passes the epithelial barrier only in very small amounts; however, if the intestinal permeability is compromised, LPS translocation into the blood stream can lead to endotoxemia (i.e., the “leaky gut” concept). Previous reports have shown that physiological factors such as strenuous physical exercise, especially under warm conditions, high-fat diet or physiological stress can elevate plasma LPS levels^43–45^. Plasma levels of LPS are challenging to measure in humans due to large fluctuations in LPS levels during the day, easy contamination of samples, and lack of sensitivity of available test kits^46,47^. Instead, plasma levels of lipopolysaccharide-binding protein (LBP) and soluble CD14 (sCD14) are recommended for the assessment of endotoxin translocation as they seem to be more stable during the day and show a minimised risk of contamination^10,48^. LBP is an acute phase lipid binding protein, synthetized by hepatocytes and intestinal epithelial cells^28^. For endotoxin recognition, LBP binds to LPS in a complex with the extracellular protein sCD14, which subsequently transfers LPS to the Toll-like receptor 4 (TLR4) eliciting a host immune response^29^. In plasma of healthy individuals, LBP is present at levels of 5 to 10 µg/ml, and levels increase approximately 20-fold during acute phase response^49^. In the current study, we showed a small but significant increase of LBP concentrations after the sauna dehydration (median of 9,407 ng/ml) compared to the control condition (median of 7,604 ng/ml). These results are still in the normal range of LBP in the circulation. Similarly, plasma LBP concentration have been previously reported to increase by 1,447 ng/ml after exertional heat stress^11,50^. In our study we observed a comparable increase of 1,803 ng/ml. This increase was accompanied by a slight, although non-significant, increase of plasma concentrations of sCD14 corresponding to 316 ng/ml after the sauna dehydration compared to the control condition. A similar increase in sCD14 concentrations (380 ng/ml) was also observed by Costa et al. and Gaskell et al. after exertional heat stress^11,50^. Hence, the translocation of bacterial products (LPS) into the blood as a result of increased intestinal permeability after sauna dehydration seems to be rather low and on similar levels as after strenuous exercise.

There are several mechanisms by which sauna dehydration might affect intestinal permeability. Sauna dehydration relies on perspiration to achieve the desired dehydration. Perspiration-induced dehydration produces a decrease in plasma volume and extracellular water which leads to hyperosmolarity. Hyperosmolarity has been reported to enhance paracellular permeability in Caco-2 cells without damaging the cell membranes via cell shrinkage^51^ and/or via hyperosmolar driven disruption of tight junctions^52^. This could explain the observed increase in intestinal permeability without intestinal damage in our study. Moreover, it has been reported, also in Caco-2 cells, that a hyperosmolar environment results in an increase of IL-8 which subsequently induces the production of IL-6^53,54^. This observation is in line with our study, as we also observed a significant increase in IL-6 and IL-8.

Another possible mechanism for an increase in permeability could be related to hyperthermia. Studies in cells have shown that hyperthermia results in an increase in permeability via disruption of the epithelial tight junctions, with more severe heat stress associated with greater permeability^55–58^. A hyperthermia-induced increase in paracellular permeability was first observed in Madin-Darby canine kidney epithelial cells exposed to a temperature of 38.3 °C or above^58^. Subsequently, Doklandy et al., using Caco-2 cells, showed a temperature-dependent increase in epithelial tight junction permeability^55^. Additionally, hyperthermia also induced an increase in cytokine levels in these cell culture models^55,57,58^. Studies in humans have shown that IL-6 seems to be increased by heat exposure only in a dose-dependent manner^8,59,60^. However, the source of IL-6 after heat exposure is still unknown^59^. In our study, the increase in IL-6 and IL-8 could be due to plasma hyperosmolarity as a result of dehydration, heat exposure and/or translocation of bacterial products, or possibly a combination of all of these factors^5,51,61–63^. We did not find any significant correlations between these factors, which might be due to a rather small number of participants and large individual differences.

In our study, male participants showed a significantly higher increase in small intestinal permeability in response to dehydration than females. The observed differences could be due to differences in body composition, fluid balance and/or hormonal regulation^32,64–67^.

One of the limitations of this study was that the impact of only sauna exposure, without dehydration, was not assessed. It would be interesting to know if the moderate increase in body temperature as part of the sauna protocol in our study by itself already resulted in an increased intestinal permeability. Another limitation is that even though this model is well tolerated by most participants, it can induce discomfort, mostly due to the water abstinence and heat stress. Indeed, we saw an increase in cortisol levels during the sauna dehydration in comparison with both the NSAID and control conditions, however, the correlation analysis did not show it to be associated with intestinal permeability. Future studies could also investigate if lower degrees of dehydration would already be sufficient to induce an increase in permeability to make the model even more applicable. In addition, future studies should also elucidate potential mechanisms behind sauna-induced dehydration, for example by investigating tight junction proteins or intestinal stem cell differentiation. Another possible mechanism which is worth exploring is the effect of sauna dehydration on ion channels, and trans epithelial transporters such as Na^+^ /H^+^ exchange (NHE), protein down-regulated in adenoma (DRA) and aquaporins. These ion channels and transporters seem to play a critical role in intestinal barrier function^68–70^ and have been linked to IBD^71–74^, diarrhoeal diseases^71,72,74^, colorectal cancer^75^, and other digestive diseases^71,72,74^. Moreover, it would be interesting to include patient groups with a potentially intestinal barrier defect such as inflammatory bowel diseases, coeliac disease or metabolic syndrome as control groups to elucidate the clinical significance of this model.

In conclusion, in the current study, we have demonstrated that sauna-induced dehydration is an accessible, non-invasive, in vivo research model to physiologically challenge intestinal permeability. This model is well tolerated by most participants and can be used to challenge the intestinal epithelial barrier with the aim to gain more insight into mechanisms of increased intestinal permeability. Sauna-induced dehydration could also be applied in clinical trials with the aim to study the effect of medical or nutritional interventions (including pre- and probiotics) intended to strengthen the gut barrier function.

## Materials and methods

### Ethical statement

The study was conducted in adherence to the ethical regulations outlined in the Helsinki declaration and its revisions. It was approved by the Central Ethical Review Board of Uppsala, Sweden on 10/01/2018 (registration number 2017/463). The study was performed at Örebro University in Örebro, Sweden, from March 2018 to June 2018. The trial has been registered at ClinicalTrials.gov on 08/08/2018 (NCT03620825). We did not consider this study as a traditional clinical trial or intervention study, as the aim was to elucidate the relationship between two physiological mechanisms, i.e., dehydration and intestinal permeability. In addition, this study did not include a patient group. This resulted in a delay in registration of this study.

### Study outcomes

The primary outcome was defined as the effect of dehydration by sauna exposure on small intestinal permeability measured as the urinary lactulose/rhamnose (L/R) secretion ratio (0–5 h). Secondary outcomes included the effect on gastroduodenal and colonic permeability as well as on biomarkers of stress and intestinal barrier function. Whole gut permeability was originally included but not assessed as it would not have contributed with relevant information.

### Sample size calculations

Own unpublished data assessing the effect of one hour of strenuous exercise on intestinal permeability compared to at rest showed a relevant difference in the lactulose/rhamnose ratio of 0.02 (mean, SD of 0.0255, unpublished). Based on this, we estimated a sample size of 13 participants with a power of 80% and a confidence interval of 95%. Taking a potential drop-out rate of 20% into account would result in a sample size of 15 participants. However, to reduce the risk of under-powering the study, we included n = 20 healthy subjects.

### Participants

Twenty healthy participants (10 females, 10 males) were recruited via advertisements at Örebro University and in social media. Subjects who expressed interest in participating in the study were further informed via email and were then invited for an explanatory meeting that took place at Örebro University. If still willing to participate, participants signed an informed consent form and were screened for eligibility for the study. Inclusion criteria were willingness to abstain from medication or probiotic products with the potential to alter gastrointestinal function during the study as well as being between 18 and 50 years of age. Reasons for exclusion were, amongst others, recent or current diseases/disorders and intake of medications. For a detailed list of exclusion criteria see Supplementary Table S6.

### Test conditions

**Figure 7:**
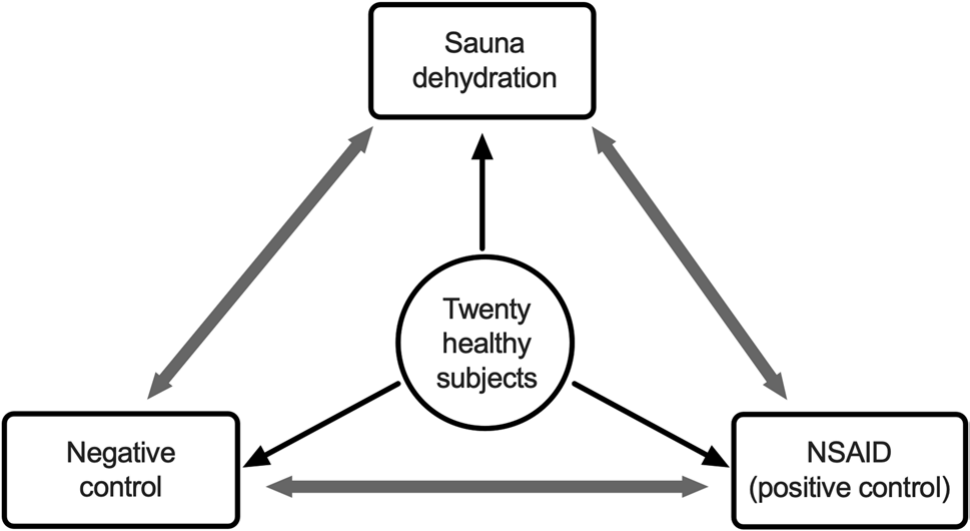
Schematic model of the study design. All 20 subjects participated in all three visits in random order: negative control, sauna dehydration and positive control. Visits were separated by a wash-out period of at least five days.

This study follwed a cross over design. All 20 healthy volunteers underwent three conditions: (1) sauna dehydration, (2) NSAID intervention (administration of indomethacin, which is known to increase small intestinal permeability^17,30–32^), and (3) control visit (see Fig. 7). The order of the test conditions was randomly assigned by the researchers based on the participants’ availability. All test conditions were performed at Örebro University and at similar hours of the day to avoid diurnal hormonal changes (Table 2) and separated by a wash-out period of at least five days. Study participants were instructed to avoid spicy foods, alcohol, drugs, artificial sugars, and strenuous exercise two days preceding and during each test day. In addition, on test days, participants were asked to avoid intake of sugars that were part of the multi-sugar intestinal permeability test (see below) and caffeine-containing foods or drinks. Participants were asked to record their food and fluid intake the evening before the first test condition, and then replicate this diet before the subsequent test conditions. Participants received a standardized meal three hours before each test, consisting of a commercial energy bar (Clif bar Chocolate chip, 257 kcal per bar, Clif Bar & Company, USA) and were then asked to fast until the end of the 5 h urine collection as part of the multi-sugar intestinal permeability test. Intake of water was allowed (ad libitum), except for the sauna condition.

**Table 2:** Study design.

	11 h before MST		3 h before MST	1 h before MST	15 min in sauna	1.5% dehydration	3% dehydration (end of sauna)	2 h after 3% dehydration
Sauna	–		S	–	T	MST, T	S,B,T	S
NSAID	NSAID 1		S	NSAID 2	–	MST	S,B	S
Control	–		S	–	–	MST	S,B	S

*MST* multi-sugar test, NSAID 1–75 mg of indomethacin, NSAID 2–50 mg of indomethacin, S-saliva sample, B-blood sample, T-body temperature.

#### Sauna visit

Before the sauna visit, participants were allowed to drink water ad libitum to achieve a euhydrated state, but were then asked to avoid water once they came to the study center. Subjects followed a dehydration scheme which comprised a sequence of 15 min periods in a dry sauna interspersed with cooling off periods of 10 min each. The sauna was maintained at circa 70 °C with lower temperatures at the lower bench and higher temperature at the higher bench, respectively. Participants were allowed to be at the height they wanted and to be in a sitting or recumbent position. Immediately after each sauna exposure, tympanic temperature was measured using a commercially available ear thermometer (Braun, ThermoScan® 7, IRT 6520). Temperature was registered at three different time points; after the first sauna period, after reaching 1.5% dehydration, and after reaching 3% dehydration. Once temperature was obtained, the subjects dried off and body weight was measured using a segmental body composition analyser (BC-418, TANITA). Participants were not allowed to drink for two hours after the multi-sugar solution was administered (at 1.5% loss of body weight by perspiration) and the sauna exposure completed (at 3% dehydration). After achieving 3% dehydration, participants dried off, and then blood samples were collected. Saliva samples were collected at the following time points: immediately after waking up (circa 3 h before the sauna exposure), when the participant had lost 3% of their body weight due to dehydration, and 2 h after the end of the sauna exposure.

#### NSAID intervention

On the evening before the NSAID intervention, circa 11 h before intake of the multi-sugar solution, participants were asked to take 75 mg of indomethacin. The next morning, three hours before the multi-sugar solution was given, participants collected the first saliva sample and consumed the provided standardized meal. An hour before intake of the sugar solution, participants took a second dose of 50 mg indomethacin. The multi-sugar solution was administered at a similar point in time as during the sauna exposure, followed by total urinary collection for 5 and 24 h. Blood and saliva samples were collected at a similar time point as during the sauna condition. During the NSAID intervention, intake of water was allowed (ad libitum).

#### Control visit

Also at the control visit, the multi-sugar solution was administered at similar time points as during the sauna dehydration condition, followed by total urinary collection for 5 and 24 h. Saliva and blood samples were collected at a similar time point as during the sauna condition. During the control visit, intake of water was allowed (ad libitum).

### In vivo intestinal permeability test (multi-sugar urinary recovery test)

Intestinal permeability was assessed by a multi-sugar urinary recovery test. For this test, 150 ml of tap water containing 1 g sucrose (Nordic sugar, Sweden), 1 g lactulose (Solactis, France), 1 g sucralose (Bulk Powders, Sweden), 1 g erythritol (Ingredi, Sweden) and 0.5 g rhamnose (BioGaia, Sweden) were orally administered after participants had emptied their bladder, followed by total urinary collection for 24 h. Urine was collected in two different fractions; fraction 1 contained the 0–5 h urinary output and fraction 2 the 5–24 h urinary output. During the first five hours of urine collection, the participants refrained from food intake and were asked to drink at least 1.5 L of tap water (during the sauna visit participants refrained from drinking until they had lost 3% of their body weight). Urine was collected by the subjects in the provided collection jars (Sarstedt, Sweden) and stored in cooling bags equipped with ice packs. After finalizing the five-hour urine collection, subjects delivered fraction 1 (0–5 h) to the university staff and continued to collect fraction 2 (5–24 h) in a second collection jar. The following day, study participants returned the second fraction to the university staff. Upon delivery of the urine samples, 1 ml of urine was centrifuged at 21,000 g for 25 min at a temperature of 4 °C. The supernatant was collected and stored at − 80 °C until analyses of the sugar proves by UPLC–MS/MS as previously described^34^.

### Salivary cortisol collection and assessment

Saliva samples were collected using Salivette collection tubes (Sarstedt, Germany). Cotton swabs were placed in the mouth of the participants for one minute. Salivettes were kept on ice until centrifugation at 3000 rpm for 5 min, which resulted in a clear supernatant of low viscosity. Saliva samples were frozen and stored at − 80 °C until analysis. Salivary cortisol concentrations were measured using a commercially available chemiluminescence immunoassay with high sensitivity (IBL-Hamburg, Germany). The intra- and inter-assay coefficients for cortisol concentration analysis were below nine percent.

### Assessment of plasma intestinal fatty acid-binding protein (I-FABP)

Plasma I-FABP concentrations were analysed using an ELISA (HK406, HycultBiotech, Uden, The Netherlands) following the manufacturer’s instructions. The detection range was specified as 47 to 3,000 pg/ml.

### Assessment of plasma liver fatty acid-binding protein (L-FABP)

Plasma L-FABP concentrations were analysed using an ELISA (HK404, HycultBiotech) following the manufacturer’s instructions. The detection range was specified as 102 to 25,000 pg/ml.

### Assessment of plasma lipopolysaccharide-binding protein (LBP)

Plasma LBP concentrations were analysed using an ELISA (HK315, HycultBiotech) following the manufacturer’s instructions. The detection range was specified as 4.4 to 50 ng/ml.

### Assessment of plasma soluble CD14 (sCD14)

Plasma sCD14 concentrations were analysed using an ELISA (HK320, HycultBiotech) following the manufacturer’s instructions. The detection range of this assay was specified to be 1.56 to 100 ng/ml.

### Assessment of plasma cytokines (IL-6, IL-8, IL-10, IFN-ɣ, TNF-α)

Plasma IL-6, IL-8, IL-10, IFN-ɣ, TNF-α concentrations, at the control visit and after sauna dehydration, were measured using a V-Plex proinflammatory panel 1, Meso Scale Discovery multi-spot assay system (MSD, Meso Scale Diagnostics, Rockville, MD) following the manufacturer’s instructions. The lower limit of quantification for this assay was specified to be of 0.063 pg/mL for IL-6, 0.591 pg/mL for IL-8, 0.298 pg/mL for IL-10, 1.76 pg/mL for IFN-ɣ and 0.690 pg/mL for TNF-α. For data analysis, values below the quantification limit were set to zero.

### Data analysis

Normality of the data sets was tested with Shapiro–Wilk test. Measurements of intestinal permeability between the test conditions were analysed using the non-parametric Friedman test. Post-hoc analyses were performed using a Dunn’s multiple comparisons test. Unpaired t test with Welch’s correction was used to analyse differences between female and male participants. All data is expressed as median and interquartile range (IQR). In order to attain a normally distributed data set, the salivary cortisol data was log2-transformed. Repeated measures ANOVA is not able to treat missing values, therefore salivary cortisol data was analysed using a mixed-effect model as established in GraphPad Prism (version 8.0). Here, Restricted Maximum Likelihood (REML) is used for data fitting and a symmetry covariance matrix and Geisser-Greenhouse correction are applied. The Tukey’s test was used to correct for multiple comparisons. Original values (not log2 transformed, median and IQR) of salivary cortisol concentrations are displayed. A two-tailed Spearman’s rank-order correlation test was used to assess correlations between variables, with adjustment for multiple comparisons using Bonferroni correction. Data was considered significant if *P* < 0.05. Correlations were made with IBM SPSS Statistics for windows, version 26 (IBM Corp., Armonk, N.Y., USA). All other statistical calculations and figures were prepared with GraphPad Prism 9.0 (GraphPad Software Incorporated, La Jolla, CA, USA).

## Supplementary Information

Supplementary Information.

## Data Availability

The datasets used and or/ analysed during the current study are available in Supplementary Table S7.
