# Supplementary material for: Sauna dehydration as a new physiological challenge model for intestinal barrier function
Source: Sci Rep. 2021 Jul 30;11:15514. doi: 10.1038/s41598-021-94814-0 (PMC8324874; doi:10.1038/s41598-021-94814-0)
Supplement: Supplementary file 1 — Supplementary Information. [file 41598_2021_94814_MOESM1_ESM.pdf]

Sauna dehydration as a new physiological challenge model for intestinal barrier function

M. Fernanda Roca Rubio[1*], Ulrika Eriksson[2], Robert J Brummer[1] and Julia König[1]

[1]Nutrition-Gut-Brain Interactions Research Centre, Faculty of Medicine and Health, School of Medical Sciences, Örebro University, 701 82 Örebro, Sweden.

[2]Man-Technology-Environment (MTM) Research Centre, School of Science and Technology, Örebro University, 701 82 Örebro, Sweden.

*Corresponding author: fernanda.roca@oru.se

Supplementary material

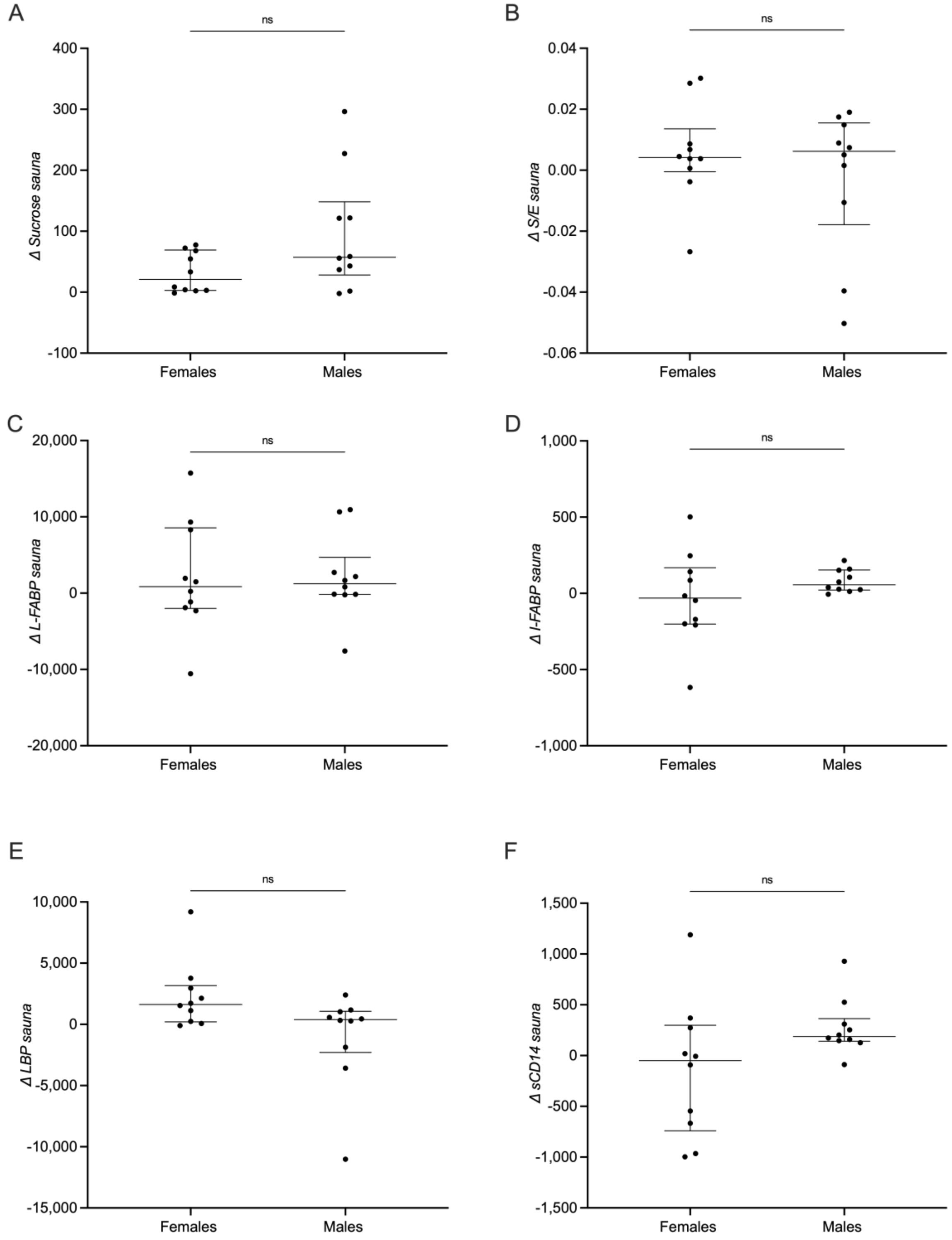

Supplementary Fig S1. Differences between female and male participants. Data is represented as Δ values: sauna value - control value. (A) Gastroduodenal permeability measured by urinary sucrose recovery (0-5h). (B) Colonic permeability measured by urinary sucralose/erythritol (S/E) excretion ratio (5-24h). (C) Plasma concentrations of intestinal fatty acid-binding protein (I-FABP). (D) Plasma concentrations of liver fatty acid-binding protein (L-FABP). (E) Plasma concentrations of lipopolysaccharide-binding protein (LBP). (F) Plasma concentrations of soluble CD14 (sCD14). The horizontal line marks the median, the vertical line spans through the interquartile range (IQR). The dots represent the individuals. ns, non-significant.

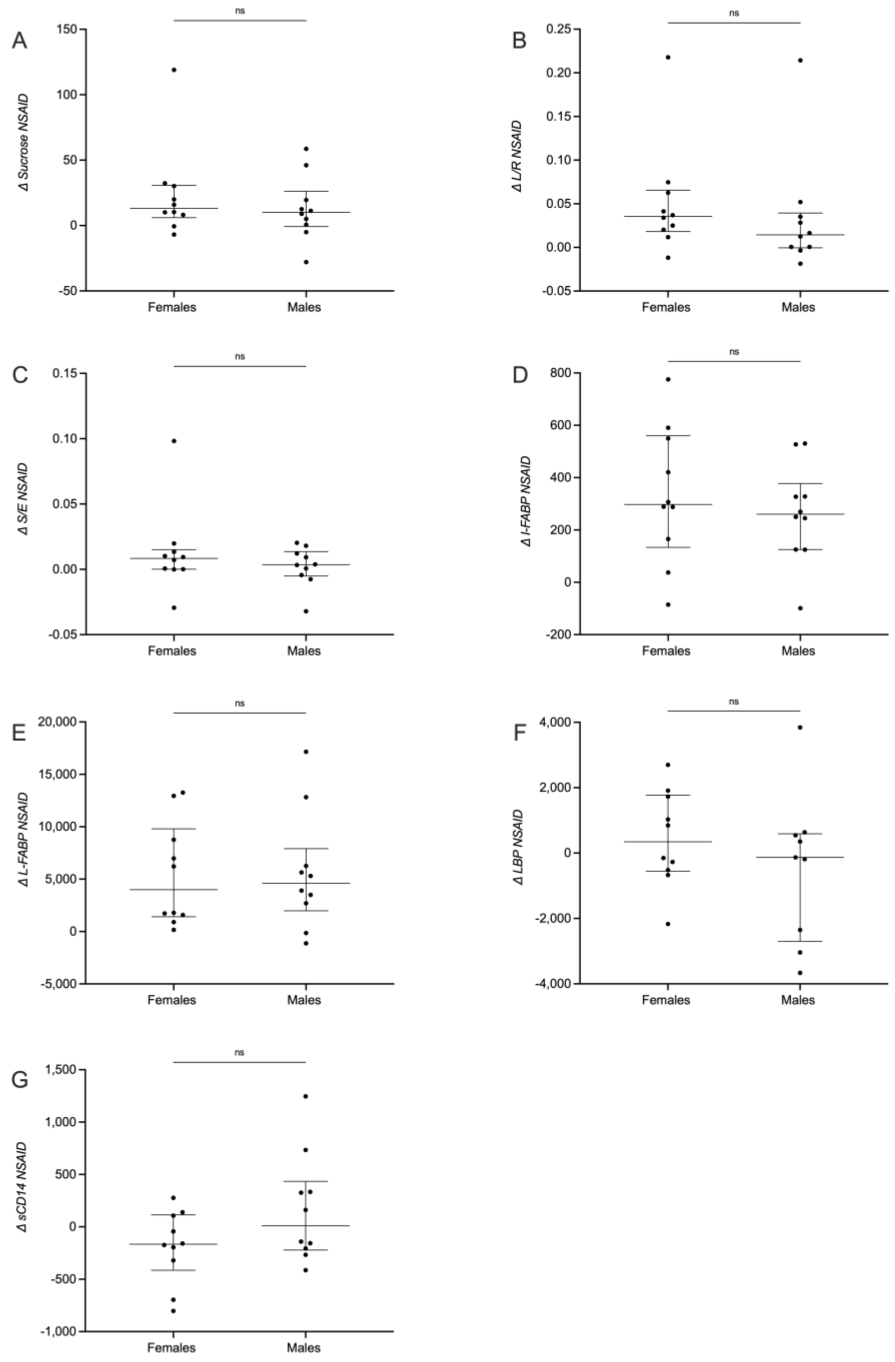

Supplementary Fig S2. Differences between female and male participants. Data is represented as Δ values: NSAID value - control value. (A) Gastroduodenal permeability measured by urinary sucrose recovery (0-5h). (B) Small intestinal permeability measured by lactulose/rhamnose (L/R) excretion ratio (0-5h). (C) Colonic permeability measured by urinary sucralose/erythritol (S/E) excretion ratio (5-24h). (D) Plasma concentrations of intestinal fatty acid-binding protein (I-FABP). (E) Plasma concentrations of liver fatty acid-binding protein (L-FABP). (F) Plasma concentrations of lipopolysaccharide-binding protein (LBP). (G) Plasma concentrations of soluble CD14 (sCD14). The horizontal line marks the median, the vertical line spans through the interquartile range (IQR). The dots represent the individuals. ns, non-significant. NSAID, nonsteroidal anti-inflammatory drug.

Supplementary Table S1. Correlations between body temperature and markers associated with intestinal permeability and cytokines after the sauna condition.

	**15 min in the sauna**	**1.5% dehydration**	**3% dehydration**
**Sucrose**	r=-0.055; p=0.858	r=-0.006; p=0.986	r=-0.634; p=0.020
**L/R**	r=-0.443; p=0.130	r=-0.075; p=0.809	r=-0.377; p=0.204
**S/E**	r=-0.234; p=0.442	r=-0.586; p=0.035	r=-0.416; p=0.157
**I-FABP**	r=0.267; p=0.378	r=-0.450; p=0.123	r=-0.270; p=0.372
**L-FABP**	r=0.341; p=0.254	r=0.030; p=0.922	r=0.275; p=0.362
**LBP**	r=0.000; p=1.000	r=-0.561; p=0.046	r=-0.094; p=0.761
**sCD14**	r=-0.286; p=0.343	r=-0.365; p=0.221	r=-0.521; p=0.068
**IL-6**	r=-0.536; p=0.059	r=0.254; p=0.402	r=0.350; p=0.241
**IL-8**	r=0.025; r=0.936	r=-0.193; r=0.527	r=-0.314; r=0.296
**IL-10**	r=-0.593; p=0.033	r=0.699; p= 0.008	r=0.611; p= 0.026
**IFN-ɣ**	r=0.407; p=0.167	r=-0.039; p=0.900	r=0.253; p=0.403
**TNF-α**	r=0.149; p=0.628	r=0.401; p=0.175	r=0.515; p=0.072

P-values smaller than 0.0015 (0.05/33, Bonferroni) were considered as significant. n=13.

Supplementary Table S2. Correlations between cytokines and L/R ratio after the sauna condition.

	**ΔL/R**
**ΔIL-6**	r=-0.114, p=0.634
**ΔIL-8**	r=-0.078, p=0.743
**ΔIL-10**	r=0.217, p=0.358
**ΔIFN-ɣ**	r=-0.483, p=0.031
**ΔTNF-α**	r=-0.065, p=0.787

P-values smaller than 0.01 (0.05/5, Bonferroni) were considered as significant. Δ: sauna value – control value.

Supplementary Table S3. Correlations between cytokines and markers associated with intestinal permeability after the sauna condition.

	**ΔIL-6**	**ΔIL-8**	**ΔIL-10**	**ΔIFN-ɣ**	**ΔTNF-α**
**ΔI-FABP**	r=0.147<br>p=0.537	r=-0.108<br>p=0.650	r=0.159<br>p=0.503	r=-0.014<br>p=0.955	r=-0.114<br>p=0.631
**ΔL-FABP**	r=0.326<br>p=0.160	r=0.183<br>p=0.439	r=-0.039<br>p=0.872	r=-0.032<br>p=0.895	r=0.125<br>p=0.600
**ΔLBP**	r=-0.118<br>p=0.620	r=-0.224<br>p=0.342	r=0.063<br>p=0.793	r=0.295<br>p=0.207	r=0.173<br>p=0.466
**ΔsCD14**	r=-0.139<br>p=0.558	r=0.573<br>p=0.008	r=0.167<br>p=0.481	r=-0.102<br>p=0.960	r=0.147<br>p=0.535

P-values smaller than 0.0025 (0.05/20, Bonferroni) were considered as significant. Δ: sauna value – control value.

Supplementary Table S4. Correlations between plasma markers associated with intestinal permeability and small bowel permeability after the sauna condition.

	**ΔL/R**
**ΔI-FABP**	r=0.144, p=0.544
**ΔL-FABP**	r=0.168, p=0.478
**ΔLBP**	r=-0.188, p=0.427
**ΔsCD14**	r=-0.053, p=0.826

P-values smaller than 0.0125 (0.05/4, Bonferroni) were considered as significant. Δ: sauna value – control value.

Supplementary Table S5. Correlations between plasma markers associated with intestinal permeability and small bowel permeability after the NSAID intervention.

	**ΔL/R**
**ΔI-FABP**	r=0.325, p=0.162
**ΔL-FABP**	r=0.296, p=0.205
**ΔLBP**	r=-0.117, p=0.662
**ΔsCD14**	r=-0.009, p=0.970

P-values smaller than 0.0125 (0.05/4, Bonferroni) were considered as significant. Δ: NSAID value – control value.

Supplementary Table S6: Exclusion criteria

- Abdominal surgery which might influence gastrointestinal function, except appendectomy and cholecystectomy
- Current diagnosis of hypertension
- Current diagnosis of psychiatric disease
- Over 100kg or with a BMI over 35
- Systemic use of steroids in the last 6 weeks
- Use of antibiotics or antimicrobial medication in the last month
- Daily usage of NSAID in the last 2 months or incidental use in the last 2 weeks prior to screening
- Usage of medications that could affect the barrier function, except oral contraceptives, during the 14 days prior to screening
- Diagnosed inflammatory gastrointestinal disease
- Known organic gastrointestinal disease (e.g. IBS, IBD, Chronic diarrhoea or constipation)
- History of or present gastrointestinal malignancy or polyposis
- Recent (gastrointestinal) infection (within last 6 months)
- Eosinophilic disorders of the gastrointestinal tract
- Current communicable disease (e.g. upper respiratory tract infection)
- Malignant disease and /or patients who are receiving systemic anti-neoplastic agents)
- Chronic neurological/neurodegenerative diseases (e.g. Parkinson´s disease, multiple sclerosis)
- Autoimmune disease and/or patients receiving immunosuppressive medications
- Major relevant allergies (e.g. food allergy, multiple allergies)
- Chronic pain syndromes (e.g. fibromyalgia)
- Chronic fatigue syndrome
- Regular use of probiotics in the last 6 weeks
- Smoking and/or chewable tobacco
- Planned changes to current diet or exercise regime
- Use of laxatives, anti-diarrhoetics, anti-cholinergics within last 4 weeks prior to screening
- Use of immunosuppressant drugs within last 4 weeks prior to screening
- Women: Pregnancy, lactation
- Abuse of alcohol or drugs
- Any disease/condition which in the investigator's opinion could interfere with the intestinal barrier function
- Any clinically significant disease/condition which in the investigator’s opinion could interfere with the results of the trial

Supplementary Table S7. Data file

Subject ID	Age (years)	sex	Weight (kg)	Height (m)	BMI (Kg/m^2)
**DH001**	23	F	66	1.7	22.8
**DH002**	36	M	71	1.75	23.2
**DH003**	27	M	72.7	1.73	24.3
**DH004**	23	F	62.3	1.68	22.1
**DH005**	26	F	60.5	1.68	21.4
**DH006**	24	M	91.1	1.97	23.5
**DH007**	38	F	54.9	1.68	19.5
**DH008**	23	F	83.8	1.74	27.7
**DH009**	24	M	86	1.76	27.8
**DH010**	24	M	86.8	1.9	24.0
**DH011**	24	F	55.8	1.71	19.1
**DH012**	28	F	56.2	1.64	20.9
**DH013**	23	F	70	1.67	25.1
**DH014**	25	F	55.6	1.65	20.4
**DH015**	29	M	86.6	1.83	25.9
**DH016**	26	M	93.2	1.78	29.4
**DH017**	21	F	54.7	1.63	20.6
**DH018**	28	M	73.7	1.75	24.1
**DH019**	38	M	78.7	1.96	20.5
**DH020**	23	M	73.9	1.92	20.0

Supplementary Table S7. Data file

Subject ID	Time to 1.5% dehydration	Time to 3% dehydration	LR ratio Control	LR ratio NSAID	LR ratio Sauna
**DH001**	02:17	04:15	0.022663301	0.240485058	0.06968805
**DH002**	01:54	04:09	0.048910912	0.049504694	0.085875299
**DH003**	01:20	02:32	0.017726847	0.034156414	0.028235052
**DH004**	01:26	03:20	0.028198687	0.048429799	0.059032706
**DH005**	02:12	04:56	0.028114844	0.069446037	0.11820283
**DH006**	01:42	03:26	0.06335837	0.277621965	0.204674793
**DH007**	02:01	03:45	0.048659192	0.036805061	0.044332504
**DH008**	02:41	04:01	0.043120221	0.054978826	0.050585837
**DH009**	02:33	03:55	0.023699692	0.052144274	0.137227047
**DH010**	01:26	02:44	0.014448519	0.06639023	0.089909562
**DH011**	01:15	02:29	0.042738077	0.117472017	0.044031096
**DH012**	01:38	03:16	0.016842798	0.079453393	0.046391352
**DH013**	01:50	03:30	0.013191827	0.038168707	0.029265536
**DH014**	01:48	03:06	0.038010442	0.074977924	0.053835164
**DH015**	01:56	03:50	0.039064986	0.039607273	0.16755477
**DH016**	01:55	03:45	0.030226382	0.026677543	0.038875839
**DH017**	01:26	02:41	0.038102144	0.072194584	0.041048926
**DH018**	01:33	03:48	0.013845337	0.026329897	0.098510469
**DH019**	01:26	03:11	0.018611224	Missing[1]	0.061806522
**DH020**	01:52	03:20	0.015324783	0.050520398	0.059325895

[1] Sample missing due to errors in the 0-5h urine collection

Supplementary Table S7. Data file

Subject ID	SE ratio Control	SE ratio Positive	SE ratio Sauna	Sucrose (µg/mL) Control	Sucrose (µg/mL) NSAID
**DH001**	0.057422654	0.028087449	0.030691988	6.782	36.972
**DH002**	0.077677904	0.045607657	0.027388584	15.562	74.212
**DH003**	0.036418981	0.040227741	0.037962128	4.158	4.824
**DH004**	0.018232394	0.028445641	0.018844607	5.388	15.486
**DH005**	0.02014852	0.118347718	0.050337505	38.954	157.916
**DH006**	0.027905059	0.037181229	0.045360725	41.944	61.508
**DH007**	0.02827853	0.028238341	0.032061244	5.854	5.34
**DH008**	0.02458753	0.044384693	0.03319868	19.79	39.818
**DH009**	0.032468191	0.033191998	0.037511229	21.648	67.78
**DH010**	0.020438656	0.038531981	0.035308843	6.158	11.27
**DH011**	0.018063901	0.031491021	0.021827917	6.386	16.734
**DH012**	0.021544492	0.030991399	0.026072632	12.216	44.584
**DH013**	0.028165888	0.028734381	0.034957665	9.6	25.576
**DH014**	0.017685782	0.024895605	0.046191956	13.308	21.504
**DH015**	0.088238331	0.080737071	0.048618285	3.958	12.948
**DH016**	0.049792612	0.045482678	0.039205884	4.27	15.574
**DH017**	0.021408802	0.021574813	0.017631293	19.76	12.85
**DH018**	0.029742195	0.033110444	0.048779095	9.718	4.662
**DH019**	0.02728297	0.047548052	0.034691825	27.928	Missing[1]
**DH020**	0.020049071	0.032196738	0.028983353	5.048	17.546

[1] Sample missing due to errors in the 0-5h urine collection

Supplementary Table S7. Data file

Subject ID	Suc (µg/ml) Sauna	I-FABP (pg/ml) Control	I-FABP (pg/ml) NSAID	I-FABP (pg/ml) Sauna	L-FABP (pg/ml) Cntrl
**DH001**	84.246	844.66	1435.34	227.908	12133.98
**DH002**	136.926	483.4913333	733.72	634.6466667	14198.19333
**DH003**	41.112	187.9326667	718.448	403.1093333	9676.386667
**DH004**	60.042	393.8866667	431.378	377.6866667	12821.05333
**DH005**	111.438	35.28466667	810.5766667	536.9526667	1722.893333
**DH006**	269.24	314.3426667	439.0126667	338.7866667	11051.16
**DH007**	39.056	184.834	472.5886667	326.6926667	10277.59333
**DH008**	22.892	166.8613333	455.518	251.9693333	1660.753333
**DH009**	143.596	188.5933333	715.5513333	263.0893333	1797.5
**DH010**	302.458	442.4873333	770.448	454.658	14907.63333
**DH011**	4.91	284.0506667	704.6906667	237.5793333	1724.233333
**DH012**	80.326	297.592	212.3306667	127.0106667	16217.40667
**DH013**	18.168	275.6093333	582.2766667	522.57	1923.893333
**DH014**	15.55	588.72	1138.526	381.4986667	15870.5
**DH015**	47.056	281.53	526.514	440.2406667	1802.3
**DH016**	62.886	292.386	417.6406667	319.976	1812.76
**DH017**	23.686	408.0366667	573.6173333	208.4293333	12883.28667
**DH018**	7.52	530.6353333	431.802	524.6493333	14379.15333
**DH019**	29.682	420.0266667	690.0286667	458.5846667	9248.753333
**DH020**	60.982	185.7946667	512.7486667	291.3406667	2042.566667

Supplementary Table S7. Data file

Subject ID	L-FABP (pg/ml) NSAID	L-FABP (pg/ml) Sauna	LBP (ng/ml) Control	LBP (ng/ml) NSAID	LBP (ng/ml) Sauna
**DH001**	18348.38667	1570.066667	6947.5	8858.333333	8665.66667
**DH002**	16893.9	15868.87	11198	8159	11644.3333
**DH003**	14980.1	11851.8	4463.333333	8305.666667	6869.5
**DH004**	14601.98	14761.61333	8111.5	5940	11883.3333
**DH005**	14670.52	11043.2	8349.333333	11048.66667	9480
**DH006**	16691.65333	13762.46667	7422.333333	8060	7751.66667
**DH007**	11184.89333	9123.386667	5946.666667	7673.333333	8075
**DH008**	10423.96667	9976.32	5167	5014	5243.33333
**DH009**	18951.06	1643.306667	11852.33333	12395	12877.3333
**DH010**	18820.84667	15723.20667	15795.66667	12129.66667	12213.6667
**DH011**	14986.88	1947.706667	5568.333333	4898.666667	5475
**DH012**	16383.83333	14314.94	10533	10014.66667	10777.3333
**DH013**	8907.4	17661.18	6848.333333	6576.333333	8384.66667
**DH014**	17445.96667	13566.47333	6451	7481.333333	9407
**DH015**	5297.026667	12754.50667	7603.5	7470	8781.5
**DH016**	1670.026667	1682.306667	4498.5	4312	4776.5
**DH017**	14610.88667	14374.63333	13564.66667	14410.66667	22753
**DH018**	13250.95333	14170.35333	10392.5	10747.66667	10967.3333
**DH019**	15517.54	1666.413333	11615.66667	9261.666667	9748.33333
**DH020**	14863.27333	12702.7	23100*	9189.333333*	12078.6667*

*Excluded data due to disproportionately high baseline values.

Supplementary Table S7. Data file

Subject ID	sCD14 (ng/ml) Control	sCD14 (ng/ml) NSAID	sCD14 (ng/ml) Sauna	IL6 (pg/ml) Control	IL6 (pg/ml) Sauna
**DH001**	2791.6	1988.68	3065.92	0.00000	1.75603
**DH002**	2097.76	2424.24	3027.08	1.48613	9.34268
**DH003**	1799.76	2534.2	2109.8	0.00000	1.81093
**DH004**	3216.4	3173.24	2250.72	0.00000	5.45587
**DH005**	3202.4	2506.76	2205.72	0.00000	1.53531
**DH006**	1672.76	2007.04	1926.12	0.00000	7.23251
**DH007**	1265	1404.64	2453.6	0.00000	3.84927
**DH008**	1840.88	1682.2	2211.08	0.00000	11.8611
**DH009**	1484.8	1344.16	1657.36	1.23306	8.63654
**DH010**	1760.24	1922.2	2285.76	0.00000	0.00000
**DH011**	1841.64	1521	1834.6	0.00000	9.24615
**DH012**	2507.68	2783.76	1840.88	0.00000	11.3204
**DH013**	2185.12	1989.68	2204.76	0.00000	12.8571
**DH014**	2373.2	2199.64	1826.92	0.00000	1.97490
**DH015**	2196.8	1930.6	2322.96	0.00000	16.2766
**DH016**	2156.44	2000.24	2303.04	0.00000	16.0891
**DH017**	1550.88	1657.2	1458.8	0.00000	2.28479
**DH018**	1794.08	1380.64	1704.8	0.00000	8.38460
**DH019**	1711.32	1504.92	1913.28	0.00000	0.00000
**DH020**	1635.96	2882.32	1794.76	4.95923	8.93160

Supplementary Table S7. Data file

Subject ID	IL8 (pg/ml) Control	IL8 (pg/ml) Sauna	IL10 (pg/ml) Control	IL10 (pg/ml) Sauna	INF-Y (pg/ml) Contro
**DH001**	5.97289	10.25407	0.00000	0.00000	6.01598
**DH002**	7.17599	9.89999	0.00000	0.00000	14.27588
**DH003**	5.27611	8.52533	0.00000	0.00000	8.00875
**DH004**	6.12601	7.49315	1.45436	0.73452	8.40092
**DH005**	9.75626	8.17493	0.00000	0.00000	5.09799
**DH006**	4.91118	9.39039	0.00000	0.00000	20.0557
**DH007**	5.60137	12.96781	0.00000	0.00000	15.78003
**DH008**	5.13942	8.33903	0.00000	0.69212	5.21781
**DH009**	6.90792	9.42978	0.00000	1.40163	8.91961
**DH010**	3.82622	7.39070	0.00000	0.00000	52.55335
**DH011**	6.49837	13.36403	0.89300	0.73205	6.97159
**DH012**	4.73128	7.23908	0.00000	0.59600	5.39184
**DH013**	5.04482	7.84199	0.00000	0.00000	28.1372
**DH014**	4.89330	5.99875	0.59600	0.00000	4.05176
**DH015**	5.13231	8.92908	0.00000	1.16735	6.95366
**DH016**	5.87487	7.97528	0.68473	0.00000	8.68434
**DH017**	6.76680	6.77033	0.59600	0.74005	22.25339
**DH018**	7.08424	7.88986	0.00000	0.00000	6.34018
**DH019**	9.17284	8.19719	0.00000	0.00000	8.24331
**DH020**	3.70251	8.97591	2.81136	0.00000	17.88639

Supplementary Table S7. Data file

Subject ID	INF-Y (pg/ml) Sauna	TNF-a (pg/ml) Control	TNF-a (pg/ml) Sauna	Temperature (Celcius) 15min
**DH001**	7.28489	1.67579	2.59273	38
**DH002**	17.48817	2.18899	2.61847	39
**DH003**	8.25536	2.12861	2.40253	39.4
**DH004**	7.75501	2.53825	2.77422	38.3
**DH005**	5.1519	2.09293	2.36822	36.5
**DH006**	13.18725	1.9019	2.19628	38
**DH007**	19.11327	1.94646	2.14463	38.9
**DH008**	7.24254	2.35388	2.96537	36.2
**DH009**	13.4917	2.29948	2.381	35.7
**DH010**	11.17918	2.66999	2.78274	Missing[2]
**DH011**	16.0437	2.21056	2.84092	36.1
**DH012**	6.66574	2.07572	2.32645	35.9
**DH013**	33.34557	3.03221	2.80965	Missing[2]
**DH014**	3.34267	2.48405	2.32638	Missing[2]
**DH015**	7.88151	1.85007	2.36099	34.6
**DH016**	9.25869	2.60118	2.43518	Missing[2]
**DH017**	35.70268	2.74706	3.02945	38.8
**DH018**	6.88151	2.82393	2.4454	Missing[2]
**DH019**	5.55953	2.44833	1.99248	Missing[2]
**DH020**	5.01457	3.3127	2.57834	Missing[2]

[2] Due to technical difficulties, temperature data of seven participants are missing from the analysis.

Supplementary Table S7. Data file

Subject ID	Temperature (Celcius) 1.5%Deh	Temperature (Celcius) 3%Deh	Salivary cortisol (nmol/L) Control Base line -2h	Salivary cortisol (nmol/L) Positive Base line -2h	Salivary cortisol (nmol/L) Sauna Base line -2h
**DH001**	39.1	36.1	11.24	5.015	9.445
**DH002**	38.8	37.3	6.51	10.775	9.26
**DH003**	38	38.6	6.735	5.98	11.78
**DH004**	39.7	39.1	7.14	2.765	4.25
**DH005**	38	36.9	7.175	1.695	3.365
**DH006**	37.9	38.6	4.33	1.865	6.055
**DH007**	37.9	37.5	6.61	8.365	9.325
**DH008**	38.1	39	22.02	5.205	1.815
**DH009**	40	37.9	5.745	12.03	5.34
**DH010**	Missing[2]	38.3	Missing[3]	0.725	2.685
**DH011**	39.6	39.5	9.94	10.68	9.375
**DH012**	39.7	38	12.425	4.52	10.17
**DH013**	Missing[2]	Missing[2]	6.53	13.025	9.41
**DH014**	Missing[2]	Missing[2]	9.425	9.745	7.96
**DH015**	38.8	38.7	15.14	18.355	12.185
**DH016**	Missing[2]	Missing[2]	3.805	4.565	3.04
**DH017**	39.5	39.5	2.555	4.41	3.08
**DH018**	Missing[2]	38.1	5.605	3.045	15.155
**DH019**	Missing[2]	37.8	3.62	5.75	1.875
**DH020**	Missing[2]	38.1	1.62	0.87	1.15

[2] Due to technical difficulties, temperature data of seven participants are missing from the analysis.

[3]Sample missing due to errors in saliva collection

Supplementary Table S7. Data file

Subject ID	Salivary cortisol (nmol/L) Control Directly after sauna 1min	Salivary cortisol (nmol/L) Positive Directly after sauna 1min	Salivary cortisol (nmol/L) Sauna Directly after sauna 1min
**DH001**	14.205	2.105	9.9
**DH002**	2.31	6.595	8.485
**DH003**	5.795	4.115	7.815
**DH004**	4.13	2.285	19.285
**DH005**	3.19	11.67	5.055
**DH006**	17.07	3.84	5.26
**DH007**	5.745	7.425	10.735
**DH008**	1.8	9.75	20.24
**DH009**	2.375	10.715	38.375
**DH010**	Missing[3]	10.12	5.825
**DH011**	10.54	9.41	6.67
**DH012**	5.86	6.425	20.52
**DH013**	8.765	8.67	19.715
**DH014**	2.71	2.015	3.355
**DH015**	4.265	4.27	42.375
**DH016**	5.045	4.065	9.205
**DH017**	12.015	10.04	12.19
**DH018**	4.465	4.19	6.11
**DH019**	13.205	9.725	10.425
**DH020**	8.635	13.35	10.53

[3]Sample missing due to errors in saliva collection

Supplementary Table S7. Data file

Subject ID	Salivary cortisol (nmol/L) Control 2h after sauna	Salivary cortisol (nmol/L) Positive 2h after sauna	Salivary cortisol (nmol/L) Sauna 2h after sauna
**DH001**	4.285	3.16	4.22
**DH002**	2.56	2.27	10.5
**DH003**	4.84	3.075	17.515
**DH004**	3.08	4.505	3.585
**DH005**	2.035	4.19	2.59
**DH006**	6.345	7.295	14.16
**DH007**	2.93	2.93	2.51
**DH008**	1.245	Missing[3]	3.86
**DH009**	1.545	3.48	9.645
**DH010**	Missing[3]	1.96	5.19
**DH011**	2.49	2.88	3.175
**DH012**	3.56	3.6	2.3
**DH013**	2.64	2.56	3.41
**DH014**	1.985	2.425	2.14
**DH015**	3.27	3.925	23.74
**DH016**	2.16	0.785	3.585
**DH017**	6.545	15.725	7.49
**DH018**	2.61	Missing[3]	5.025
**DH019**	4.195	3.69	Missing[3]
**DH020**	3.12	3.75	3.6

[3]Sample missing due to errors in saliva collection
